# Miscellaneous Surgical Essays

**Published:** 1781-09

**Authors:** 


II. *Miscellaneous surgical Essays. By* J. L. Schmucker. *(Continued from page* 116).

XXV. *AN account of an inveterate lues venerea, by* Mr. Muller.—The principal complaint in this case was a caries of the cranium. The

The diſeaſed portion of bone was removed by four applications of the trepan. In about a fortnight after the operation the patient began to take the bark, to which mercury was afterwards added, and by theſe means he recovered.

XXVI. *Caſe of a violent concuſſion of the brain, by* Mr. Klemann.—In this patient the ſymptoms that commonly attend caſes of this ſort were accompanied with a violent delirium, which had continued twenty-ſeven days, when our author reſolved to cover the whole head with a bliſter. The good effects of this application were ſoon perceptible, and the patient got well.

XXVII. *Caſe of a concuſſion of the brain, by* Mr. Creuzwieſer.—In this caſe after repeated bleedings, glyſters, bliſters, and cold applications to the head had proved ineffectual, recourſe was had to emetic tartar. Fifteen grains of it proved of no uſe, but when twenty-five grains of it were adminiſtered, a violent mucous vomiting was excited, which immediately relieved the patient, who afterwards recovered.

XXVIII. *Of the cure of a large ſwelling of the thyroid gland, by* Mr. Sellie.—In this caſe the tumour was gradually conſumed by ſuppuration.

XXIX. *A caſe of hernia, by* Mr. Weidele.—The ſcrotum, inteſtine, teſticle, and ſpermatic

 chord, chord, in this case, were mortified. All these parts were separated by the knife, and the author had the satisfaction to see his patient recover; but the fœces continued to pass through the wound.

XXX. *An account of a singular spasmodic affection of the stomach, by* Mr. Hemann.—The complaint here spoken of, after resisting a variety of remedies, was at length relieved by the patient's drinking a glass of cold water every morning.

XXXI. *A case of ischuria, by* Mr. Hagen.—The patient whose case is here described was in the fourth month of her pregnancy, and the ischuria was occasioned by a falling down of the upper part of the vagina. The prolapsus however gradually disappeared as the uterus increased in bulk, and the patient suffered no other inconvenience than that of being obliged to submit occasionally to the introduction of a catheter.

XXXII. *A case of hernia attended with a singular circumstance, by* Mr. Lange.—The patient who is the subject of this paper voided his fœces through a wound which remained after a strangulated hernia that had mortified, and nearly proved fatal. By carrying a heavy burthen a considerable portion of intestine was forced through this opening, and could not possibly be reduced withwithout dilating this artificial anus. This was accordingly done, and the gut was then eaſily reſtored to its natural ſituation, a conſiderable quantity of blood being diſcharged from its inner coat during the operation.

XXXIII. *Of a venereal ulcer, by* the ſame.—We have here an account of a painful ulcer of the cheek, that had deſtroyed almoſt the whole of the parotid gland before it was ſuſpected to be owing to a venereal taint. The excretory duct of the gland being eroded, the ſaliva was to be ſeen trickling from different parts of the wound. In this ſtate Mr. Lange adminiſtered a ſolution of ſublimate internally, and applied red precipitate to the ulcer. Theſe remedies produced the deſired effect. The ſaliva continued for ſome time to be diſcharged through a fiſtulous opening, but this was at length healed by touching it with *lapis infernalis*, and applying compreſſes to it according to the method recommended by M. Louis.

XXXIV. *An account of a venereal palſy of the arm, by* the ſame.—The cure of this patient was effected by giving the ſublimate, camphor, and a decoction of ſaſſafras internally, and applying at the ſame time *linimentum ſapcnaceum* to the diſeaſed arm.

XXXV. *An account of the extirpation of a polypus from the rectum, by* the ſame.—This was done by means of a ligature. The polypus, which was about the ſize of a walnut, ſeparated in ſix days without pain.

XXXVI. *Of the fatal effects of an immoderate uſe of brandy, by* Mr. Völker.—The effects produced in this caſe were indigeſtion, loſs of appetite, vomiting after eating, obſtinate coſtiveneſs, pain under the ſhort ribs, anxiety, and at laſt death. Upon diſſection the liver was found hard and conſiderably enlarged, the ſtomach nearly of a cartilaginous conſiſtence and greatly contracted, and the meſenteric glands indurated.

XXXVII. *Caſe of an aneuriſm of the pulmonary artery, by* Mr. Eiſenſchmidt.—At firſt this diſeaſe was attended only with dyſpnœa, but after a time the third and fourth true ribs were elevated, and at length the patient was ſuddenly ſuffocated. Upon diſſection the aneuriſmal ſac was found to contain three pounds of blood and the two ribs were carious.

XXXVIII. *Caſe of a gun-ſhot wound of the throat, by* Mr. Rüdiger.—In this caſe the ball paſſed obliquely in under the left ſide of the cricoid cartilage, lacerated the membranous part of the aſpera arteria, and came out between the maſtoid proprocess and the upper jaw. The patient was unable to ſwallow for ſome days after the accident, ſo that probably the œſophagus alſo was wounded. At firſt, though he coughed up a conſiderable quantity of blood, and afterwards blood mixed with pus, but at length the wound healed and the patient recovered.

XXXIX. *Caſe of a luxation of the os femoris, by* Mr. Sonderſhof.—In this caſe the bone was diſlocated upwards, and reduced on the nineteenth day after the accident.

XL. *Caſe of a luxation of the os humeri, by* Mr. Schmucker.—In this patient the bone had been diſlocated upwards of three months, when the reduction was attempted by our author. The extenſion was made ſlowly and by intervals, in order to give the contracted muſcles time to elongate, and in about three quarters of an hour this method had the deſired ſucceſs.

XLI. *An account of a caſe in which the inteſtine was perforated by a worm, by* Mr. Luducke.—The death of this patient was preceded by jaundice, hectic fever, and a painful ſwelling in the right hypochondrium. Upon diſſection the thoracic and abdominal viſcera were found greatly diſeaſed, and the colon perforated by a worm.

XLII. *An account of an hydrocele of the tunica vaginalis ſucceſsfully treated, by* Mr. Schwindt.— The cure in this caſe is ſaid to have been effected by repeatedly expoſing the ſcrotum to the vapour of vinegar, and afterwards applying to it compreſſes moiſtened with the ſame liquor. The ſcrotum was ſuſpended by a bag truſs, and in a few days the ſwelling entirely diſappeared. The complaint returned four times, but was always removed by the ſame method, and the patient, we are told, has ſince remained perfectly free from it.

XLIII. *An account of an inveterate fiſtula, by* Mr. Seeliger.—This fiſtula is the conſequence of a gun-ſhot wound of twenty years ſtanding. The ball paſſed in an oblique direction between the muſcles of the abdomen and peritonæum from the left groin to the os ilii. The patient enjoyed a tolerable ſtate of health, while the diſcharge from the wound continued, but at times this diſcharge is ſuſpended, and then he is conſtantly troubled with a variety of diſtreſſing ſymptoms till it is reſtored again, which generally happens in a few days, the paroxyſm terminating in a critical diſcharge by ſweat and urine. Theſe ſymptoms are attributed by Mr. Seeliger to an abſorption of the pus. He ob-

ſerves

ſerves likewiſe that the patient has a conſtant exſudation of an acrid humour through the ſkin of the anus, which ſeems to have a ſalutary effect.

XLIV. *An account of the efficacy of the ſemina ſabadillæ as a cure for the tænia, by* the ſame.—In a caſe of this ſort our author ſucceeded in the cure by means of this medicine after every other remedy had proved ineffectual. He gave half a drachm of the ſeeds in powder, mixed with honey, every morning, and every fifth day adminiſtered a draſtic purge. In fourteen days the worm was completely expelled. Mr. Seeliger adds, that he has likewiſe found the powder of theſe ſeeds very efficacious in deſtroying bugs in houſhold furniture.

XLV. *Caſe of a gun-ſhot wound in the head, by* Mr. Rüdiger.—In this patient part of the outer table of the os frontis was carried away, and the inner table depreſſed by the ball. At firſt no particular ſymptoms were excited, and our author contented himſelf with the uſe of cold applications to the wound; but at the end of a fortnight the patient began to complain of pain in his head and other ſymptoms, which indicated the uſe of the trepan. The inſtrument was applied in three places. The dura mater apappeared much inflamed, and under it a fluctuation was perceptible. On puncturing this membrane, pus and coagulated blood were discharged, and the patient found himfelf fomewhat relieved, but two days after died lethargic. On diffection the veffels of the dura mater were found præternaturally diftended with blood, and under this membrane was a great quantity of pus, a confiderable portion of the brain having fuppurated.

XLVI. *Cafe of a gun-fhot wound, by* Mr. Otto. —In this cafe a flight fiffure was difcovered in the left parietal bone, and a fmall part of the bone was depreffed. On the fifth day after the accident the patient complained of an acute pain in the head, and the trepan was applied in two places. The depreffed fracture and feveral other pieces of the bone being by this means removed, the patient found himfelf better; but on the feventh day the head-ach returned again. On the 23d the pulfe quickened, the patient became comatous, and the wound affumed an unfavourable afpect. Shortly after this the patient died, and upon diffection the brain and its membranes were found in a ftate of fuppuration.

XLVII. *A cafe*

XLVII. *A case of hernia humoralis, by* Mr. Grofs.—This complaint was the confequence of a dyfentery. A fluctuation being felt in the tefticle, an incifion was made into the fcrotum, and the matter evacuated. Upon examination the tefticle itfelf appeared to be found, but its tunica vaginalis was in a difeafed ftate. This cafe, and we could add to it another fimilar one from our own obfervation, feems to prove, that in a hernia humoralis the tunica vaginalis, and not the teftis, is the feat of the difeafe.

XLVIII. *An account of a cancerous tumor fuccefsfully extirpated, by* Mr. Schwindt.—The tumor here fpoken of was fituated under the left cheek. It was very hard, and difcharged an acrid ftinking ichor.

XLIX. *An account of a gun-fhot wound in the arm, by* Mr. Sonderfhof.—This cafe proved fatal, which might probably be owing to the neglect of amputation in the beginning.

L. *An account of a large fteatomatous tumour, by* Mr. Devrient.—This tumour was fituated on the infide of the thigh. A fluctuation being felt in it, it was opened, and found to contain an ichorous matter. The patient died confumptive, and upon examination the fac was found to extend under the femoral artery.

LI. *An account of a total defect of the uterus and vagina, by* Mr. Meyer.—This obfervation was made in a dead fubject. No appearance of a vagina or uterus was to be found; in their ftead there was only a loofe cellular membrane that arofe from the peritonæum. The ovaria were nearly in a natural ftate; the labia and clitoris fmall; and the nymphæ wholly deficient.

